# Fish Traders as Key Actors in Fisheries: Gender and Adaptive Management

**DOI:** 10.1007/s13280-013-0451-1

**Published:** 2013-11-09

**Authors:** Sara Fröcklin, Maricela de la Torre-Castro, Lars Lindström, Narriman S. Jiddawi

**Affiliations:** 1 Department of Ecology, Environment and Plant Sciences, Stockholm University, 106 91 Stockholm, Sweden; 2 Department of Physical Geography and Quaternary Geology, Stockholm University, 106 91 Stockholm, Sweden; 3 Department of Political Science, Stockholm University, 106 91 Stockholm, Sweden; 4 Institute of Marine Sciences, University of Dar es Salaam, Mizingani Rd., PO Box 668, Zanzibar, Tanzania

**Keywords:** Fish value chain, Fish market, Gender analysis, Middlemen, Small-scale fisheries, Zanzibar

## Abstract

This paper fills an important gap towards adaptive management of small-scale fisheries by analyzing the gender dimension of fish trade in Zanzibar, Tanzania. We hypothesize that gender-based differences are present in the fish value chain and to test the hypothesis interviews were performed to analyze: (i) markets, customers, and mobility, (ii) material and economic resources, (iii) traded fish species, (iv) contacts and organizations, and (v) perceptions and experiences. Additionally, management documents were analyzed to examine the degree to which gender is considered. Results show that women traders had less access to social and economic resources, profitable markets, and high-value fish, which resulted in lower income. These gender inequalities are linked, among others, to women’s reproductive roles such as childcare and household responsibilities. Formal fisheries management was found to be gender insensitive, showing how a crucial feedback element of adaptive management is missing in Zanzibar’s management system, i.e., knowledge about key actors, their needs and challenges.

**Electronic supplementary material:**

The online version of this article (doi:10.1007/s13280-013-0451-1) contains supplementary material, which is available to authorized users.

## Introduction

Women have an important role in world fisheries (Bennett 2005), but traditionally, fisheries have been associated with men (Davis and Gerrard 2000; Williams et al. 2005; Samuel 2007; Williams 2008; Choo et al. 2008) with focus primarily on capture fisheries, rather than women-dominated pre- and post-harvest activities, e.g., finance, processing, and marketing the catch (Weeratunge et al. 2010; Harper et al. 2013). Women also perform diverse unpaid tasks such as mending nets, collecting bait, preparing food for fishers, and keeping accounts, which are unacknowledged or undercounted as employment (Williams et al. 2005; FAO 2006; Williams 2008). In addition, women’s gleaning of invertebrates and near shore fishing is largely undervalued and almost invisible in management plans and fisheries statistics. In spite of women’s central role, a number of factors reduce their access to fisheries resources and assets. These include traditional beliefs, norms and laws, which in turn confine them to the lowest end of fish value chains (FAO 2006; Porter 2006). Women’s relatively limited participation in fish value chains further results in gender disparities in terms of income, and often in women being entrenched in poverty, as for example, in West African fisheries (FAO 2008). It is, however, important to remember that in most developing countries men also struggle to make a living, and fisheries have often been described as a last resort occupation (Allison and Ellis 2001). However, since women are associated with reproductive work, gender inequalities in access to fisheries resources affect not only the livelihoods of women, but also the entire household (Weeratunge et al. 2010).

The importance of including gender aspects in research and policy is recognized, but commonly overlooked in the fisheries and aquaculture sectors (Tietze 1995; Diamond et al. 2003; Fröcklin et al. 2012; Harper et al. 2013). Although there are valuable studies regarding women’s role in fisheries, gender-disaggregated data on fishing activities are often absent (e.g., Weeratunge et al. 2010; Harper et al. 2013), which hinders robust comparisons and comprehensive gender analysis much needed for improved fisheries management and policy (de la Torre-Castro et al., unpublished). Further, the importance of gender aspects in adaptive fisheries management, i.e., an integrated, multidisciplinary approach based on learning-by-doing (Holling 1978; Walters 1986; Lee 1993; Gunderson et al. 1995), has not to our knowledge been addressed. One of the key elements in adaptive management is the iterative link between knowledge and action, and the need for responsive institutions. In an attempt to fill this knowledge gap we focus on the growing enterprise of fish trade in Zanzibar (Unguja Island), Tanzania, from a gender perspective and further link the findings to adaptive management aspects.

In much of Africa, women are actively involved in trade with both fish and other commodities (Walker 2001, 2002; ICSF 2002; Madanda 2003). However, in for example, the Lake Victoria fishery men tend to control the profitable large scale operations of high-value fish, while most women focus on the local market and low-value fish (Lwenya and Abila 2001). In Zanzibar, fish trade has traditionally been dominated by men but over the last years women traders (*Mama Karangas* in Swahili) have become a common sight at the local fish markets, which give rise to the following questions: What are the underlying reasons for the recent increase in women traders? Has the increase in women challenged traditional gender roles and led to women’s empowerment such as improved decision making power in the household and/or in the society at large? Is the management system accounting for gender differences in Zanzibar? We further argue that in order for fisheries management to be adaptive, knowledge about both ecological and social spheres is needed. Thus, this study fills an important gap towards adaptive fisheries management in Zanzibar by acknowledging the role of both men *and* women traders, but also their needs and challenges in the fish value chain.

## Materials and Methods

The research was undertaken in Zanzibar (Unguja Island) in March and July 2012; during these periods semi-structured interviews with men (*n* = 21) and women (*n* = 21) traders, as well as observations, at ten different fish markets took place. In addition, we analyzed documents from the Ministry of Livestock and Fisheries, with its subdivisions: Department of Fisheries Development DFD and Department of Marine Resources DMR (the institution responsible for marine resource management), to investigate to what extent gender is considered in fisheries management. Also, price information on traded fish species was collected from the monitoring agents (*Bwana Dikos* in Swahili) directly at the auction at each of the study sites.

### Study Area

**Fig. 1:**
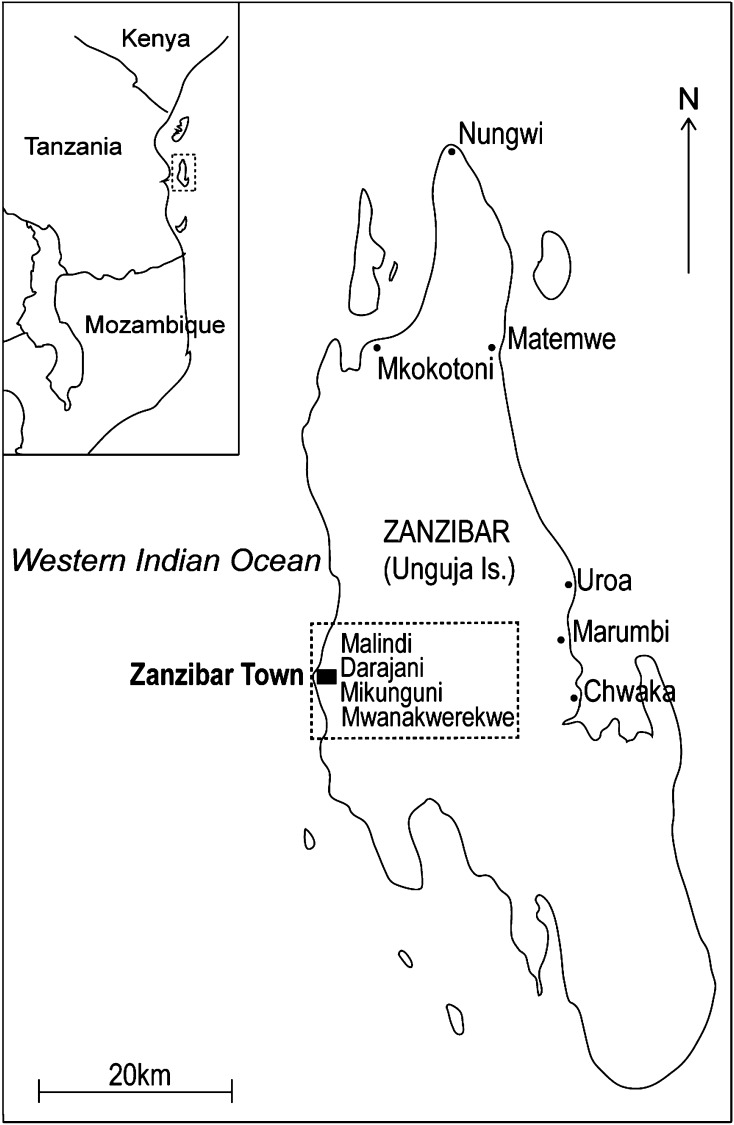
Map of Zanzibar (Unguja Island) 6°8′0′′S, 39°19′0′′E, with studied fish markets (*n* = 10). Village markets are shown by *small black dots* (*n* = 6) and Town markets are found within a *dotted line* (*n* = 4)

Zanzibar is located in the Western Indian Ocean, about 40 km off the coast of mainland Tanzania (Fig. 1). It comprises a number of small islands and two large ones; Unguja and Pemba, and is inhabited by about 1 000 000 people (Ruitenbeek et al. 2005). This study focuses on Unguja Island, hereafter referred to as Zanzibar. Fishing activities have historically provided some of the most important livelihoods and assets on the Island, thus contributing to the formal and informal economic sectors. Fish is also a relatively cheap and accessible source of protein for Zanzibar’s mainly low income households (Jiddawi and Öhman 2002), and a popular food item among tourists. Thus, the importance of fisheries based livelihoods has increased with more people engaged in fishing (Jiddawi and Khatib 2007) and fish trade-related activities.

The fish value chain in Zanzibar is rather simple; most of the sector is artisanal and characterized by non-selective fishing and the use of traditional gears and equipment (Jiddawi 2012). Fish catches are landed at sites officially selected by the DFD/DMR in Zanzibar, and monitored by an employee, the *Bwana Diko*, of the DFD (de la Torre-Castro 2006). The monitoring agents report the number of fish landed, weight and price. Fish are sold in a number of ways by the fishers, but often to traders via auctions held at the local fish markets (the local market place generally consists of brick buildings or open constructions that lack cold storage facilities except for the two main markets in Zanzibar Town) (de la Torre-Castro 2012; Thyresson et al. 2013). Traders then sell the fish, processed (salted, sun-dried, or smoked) or fresh (whole or filleted), to local customers, hotels and restaurants or via another auction. Lobsters and shrimps are, however, mainly traded directly between fishermen and men traders. Trading activities in Zanzibar involve a wide range of actors operating at different scales (Thyresson et al. 2011; Eriksson et al. 2012), and has by tradition been dominated by men or so-called “middlemen” (Crona et al. 2010), i.e., intermediaries buying the fish for further resell; they can operate by direct contact with the fishers or by buying the products at the local fish market. Middlemen may lease fishing equipment (e.g., boats, gears, and engines) (Jiddawi and Öhman 2002) or provide fishermen with credit. However, only a small proportion of Zanzibar’s fish traders have the resources to supply fishermen with equipment, boats or credit and are thus not involved in these kinds of agreements. Instead, many traders buy and sell fish independently. In this study we focus on small-scale traders operating at the local markets; to acknowledge the gender perspective we use the more suitable and gender neutral term “fish trader” instead of “middlemen”.

### Interviews

Interviews were held with 21 women (age 20–55) and 21 men (age 27–78) fish traders at ten different fish markets around the Island; Chwaka, Darajani, Malindi, Marumbi, Matemwe, Mikunguni, Mkokotoni, Mwanakwerekwe, Nungwi, and Uroa (Fig. 1). The sites were selected based on the local knowledge of scientists at the Institute of Marine Science (IMS), University of Dar es Salaam (UDSM), to cover markets of different character (i.e., men/women-dominated, high- vs. low-value fish species, and tourism influences, Table 1). The value of each species was classified according to price per kg; high value >3 US dollar (USD), medium value ≈3 USD, and low value <3 USD. Fish markets per se were not set as a unit of analysis but rather as a way to capture general differences between men and women according to the components analyzed. The targeted respondents were fish traders who regularly purchased fish at the auction and then sold it further via another auction, fish market, directly to local customers, or to hotels and restaurants. Depending on market characteristics the number of women and men interviewed at each market varied, however, summing up to a total number of 21 men and 21 women respondents (Table 1). A semi-structured interview following Kvale (1994) and Denscombe (2007) was applied to solicit information about: (i) markets, customers, and mobility, (ii) material and economic resources including initial capital, daily income, and assets (e.g., equipment), (iii) traded species, (iv) contacts and organizations with focus on membership in fish trading organizations, fishing committees, cooperatives, and contact with DFD/DMR, and (v) perceptions and experiences to examine life improvements, possible effects on a community level, equality and capacity to cope with fluctuating markets (Electronic Supplementary Material, Appendix S1). These components were developed and chosen based on the literature (e.g., Lwenya and Abila 2001; Medard et al. 2002; Weeratunge et al. 2010) and our own experiences working with coastal livelihoods in East Africa. The components provide a comprehensive picture of fish trade and help analyze the way in which fish trade and commodity flows are interwoven in wider social, cultural, economic, and environmental structures and processes. The interviewer introduced the theme and the respondents were able to speak freely on each topic. The semi-structured interview was chosen since it is one of the most powerful methods, allowing flexibility to probe for answers, follow-up the original questions and pursue new lines of questions (e.g., Kvale 1994; Denscombe 2007). Respondents were selected in consultation with the *Bwana Diko* at each fish market and according to availability and willingness to participate in the study. As there is no information on the total number of traders in Zanzibar the representativeness of the sample is difficult to assess. The interviews lasted from 40 min to 1 h and took place at the fish market or close to the respondent’s house. All interviews were performed by the same researcher and trained assistant from IMS, the latter with Swahili as mother tongue, and all interviews were recorded for further analysis. About 6 to 10 days were spent at each fish market, which helped to establish a better understanding about the activity through observations and conversations with other fish traders, *Bwana Dikos* and local people.

**Table 1:** Market characteristics including men/women dominated, fish value (high, medium, low), relative closeness to hotels/restaurants and the number of men and women interviewed at each market

Market	Men/women-dominated	High-value fish Medium Low-value fish	Relative tourism influences (hotels/restaurants close to the market)	No. of men interviewed (*n* = 21)	No. of women interviewed (*n* = 21)
Chwaka	M	Mixed	Medium	2	1
Darajani	M	Mixed	High	2	1
Malindi	M	Mixed	High	2	2
Marumbi	W	Low to medium	Medium	1	2
Matemwe	M	Mixed	High	3	0
Mikunguni	W	Low to medium	Absent	0	4
Mkokotoni	M	Mixed	Absent	2	2
Mwanakwerekwe	W	Low to medium	Absent	1	4
Nungwi	M	Mixed	High	6	2
Uroa	M	Mixed	High	2	3

### Data Analysis

The respondents’ answers were organized, transcribed, and categorized by using the pre-determined components. The data were then analyzed according to the categories to provide a comprehensive picture of the gender dimensions of fish trade. Also, familiarity with the location and community enabled us to grasp wider social issues and further reduced the interviewer effect. Text analysis of the fisheries acts and management plans was done with focus on the inclusion of gender.

## Results

### Increase in Women Traders—What is the Underlying Reason?

As mentioned previously fish trade in Zanzibar has traditionally been associated with men, which is reflected in the average number of years men and women traders had been active. Whereas the men had been involved in trading activities for on average 18 years, the number for women was nine. Based on observations and discussions with researchers at IMS, the number of women entering local fish markets has increased over the last years. The respondents reported that this increase was due to the lack of alternative economic activities and the need for all family members to contribute to household income.

### Characteristics of Fish Traders and General Work Situation

The educational level among interviewed traders was generally quite low; however, more women than men had finished primary and secondary school and a complete lack of formal education was more prevalent among men. Although women in the present study showed higher educational levels, few women traders had managed to start-up the business themselves. Instead, the majority were initially introduced and trained by husbands, friends or relatives although once introduced most women traders continued on their own. The few men who received assistance during auctions did so as a way to increase the business, or to reduce work effort by hiring people to transport and sell the fish. However, it requires economic resources and did not occur on daily basis. Women were instead in need of support in order to obtain time to continue their reproductive work in addition to productive work, and not as a means to increase profit.

Although men and women traders spent an equal amount of time (about 8 h per day) on fish trade, women’s responsibility for household duties and childcare resulted in much longer workdays. Men traders were generally present at the fish market hours before the auction started, and consequently used that “free time” to socialize with other traders, while most women showed up just in time for the auction and left immediately after it was finished. In addition to fish trade, 33 % of the women and 19 % of the men were engaged in other economic activities such as selling food and soap or agriculture to make ends meet.

### Fish Markets, Customers, and Mobility

**Fig. 2:**
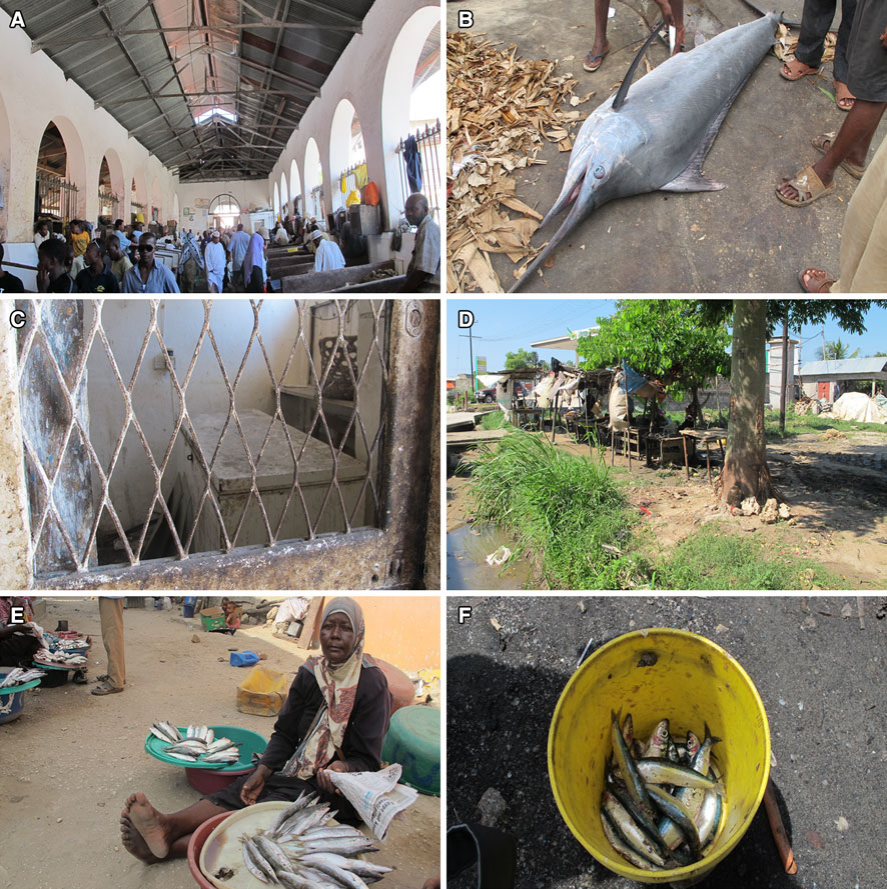
Photos showing **a** a men-dominated market in Zanzibar Town with **b** high-value fish species and **c** better access to infrastructure; **d** a women-dominated market in a suburb to Zanzibar Town with **e** low-value fish and **f** no or little access to infrastructure such as cold storing facilities. Photos: S Fröcklin

**Fig. 3:**
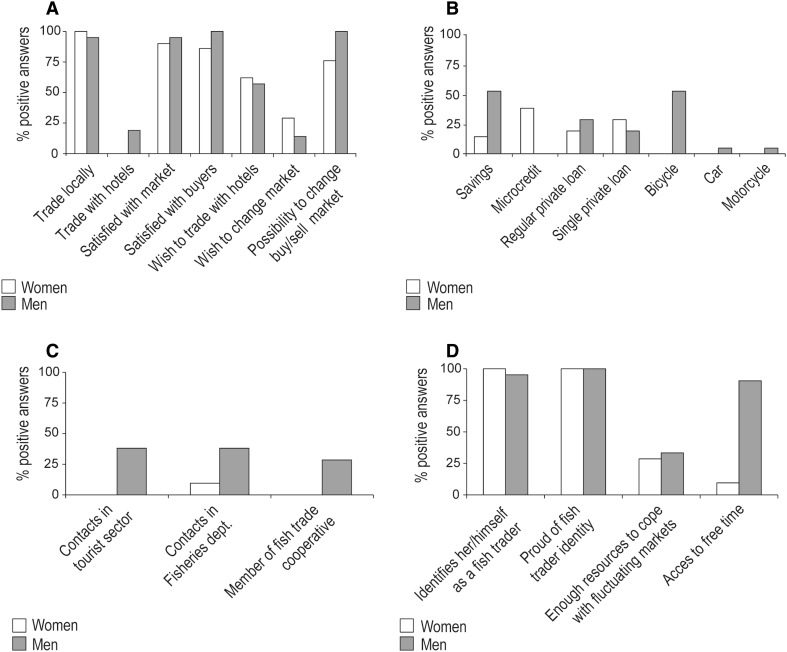
Summary of gender inequalities in **a** markets, customers, and mobility; **b** material and economic resources; **c** contacts and organizations; and **d** perceptions and experiences (percent positive answers based on 21 women and 21 men)

Both men and women generally bought fish at the same auction every day, which was reported to be due to familiarity, not only with the auction place, but also with other fish traders and fishers. Quality and availability of fish, price, and safety were reported to be less important and clearly not as important as having well established contacts. Some women mentioned the relative closeness between household and market as an important factor, which was again explained by commitment to household responsibilities. In terms of marketing, many women traders were operating by the road in a suburb to Zanzibar Town. However, they were obliged to pay a daily fee of 1 USD to a private landowner, apart from the mandatory 50 cents to the Municipality, just to access a small piece of land. Contrary to women traders men had more options in terms of fish marketing; some were selling from bicycles in Zanzibar Town which require no fees, while a few were involved in trade within the tourism industry. However, some men reported that hotels do not always pay traders at once thus only the traders with high financial capital are able to maintain a continuous trade while waiting for payment. In addition, many men were operating inside the main market buildings in Town, which offer a greater variety of customers and better profits due to higher prices (Figs. 2, 3a). Also, a typical strategy among men traders to increase the profit was to buy fish to a cheaper price at a village market, and then sell it to a higher price at one of the main markets in Zanzibar Town. The current price of renting a table inside the main market building was 50 cents per day, which is less than half the price compared to the women selling in the suburb. The main markets do not only offer a variety of customers but also cold storing facilities at a price of 20 cents per kilogram fish, which helps to preserve the fish longer and reduces a potential loss. Currently, no women rented tables inside the main markets. However, improved access to the main markets was requested by most women but regardless of free spots they reported to lack the right contacts within the Municipality and the market itself. Also, a common statement among men traders was that women should not interact with men inside the main markets, which was based on social and traditional norms and values about what a “respectable” woman should or should not do.

To access the fish markets most traders used the local buses (*Dala dala* in Swahili) (Electronic Supplementary Material, Table S2). However, 11 of the 21 men owned a bicycle, one owned a car and another one a motorcycle (Fig. 3b). Having access to own transport means makes the traders more flexible on both a temporal and spatial scale. None of the women had access to any of these assets. To cope with current market changes women borrowed money, joined other traders so as to increase the total capital, engaged in other activities to increase income or simply bought less fish. Despite a potential decline in fish landings most women stated that they would or could not change market. Instead, they would return home, empty-handed, and do something else until the market improved. On the contrary, all men reported to have the possibility to change market (Fig. 3a), and most would do so if necessary. Only a few would borrow money, use savings, or engage in other activities to keep the business going. A few men said that they would rest at home since “no market is bad for long”.

### Material and Economic Resources

#### Capital and Income

To engage in fish trade a start-up capital is needed and fish traders were thus asked to state the source of their initial funding. The three main sources included microcredit, savings, and money lent (often interest-free) by friends or relatives. Most of the women traders (86 %) used borrowed money to start-up their business and a little less than half was involved in microcredit (mainly women from Zanzibar Town) or had taken a single loan from their husband or other relative, while the rest borrowed money on a daily basis from a wealthy man in their village. Only three of the 21 women used savings. In comparison, more than half of the men used savings, some took private loans on regular basis, and just a few had taken a single loan (Fig. 3b). The capital used for fish trade varied greatly between men and women. Men purchased fish for about 64 USD at a 13 USD profit per day (median value), whereas women used approximately 48 USD at a 6 USD profit per day (median value). Daily capital used was more or less constant for both men and women, i.e., money generated was always re-invested in a “special pot” used for fish trade only. The money left after removing additional expenses such as transport, frying oil, firewood and ice, and after re-investing in the pot, was considered as income.

#### Assets

There are a number of necessary and supplementary items linked to fish trading activities such as cell phones to communicate with buyers, fish traders, or the auctioneer, and freezers to preserve fish that is not sold. About half of the men and women traders had access to phones and among the remaining traders this item was highly requested. Less than half of the men and women owned a freezer. Thus, in an attempt to preserve fish most traders bought ice on daily basis. Hitherto, men possessed greater access to freezers due to their rented tables at the main markets.

### Traded Species

**Fig. 4:**
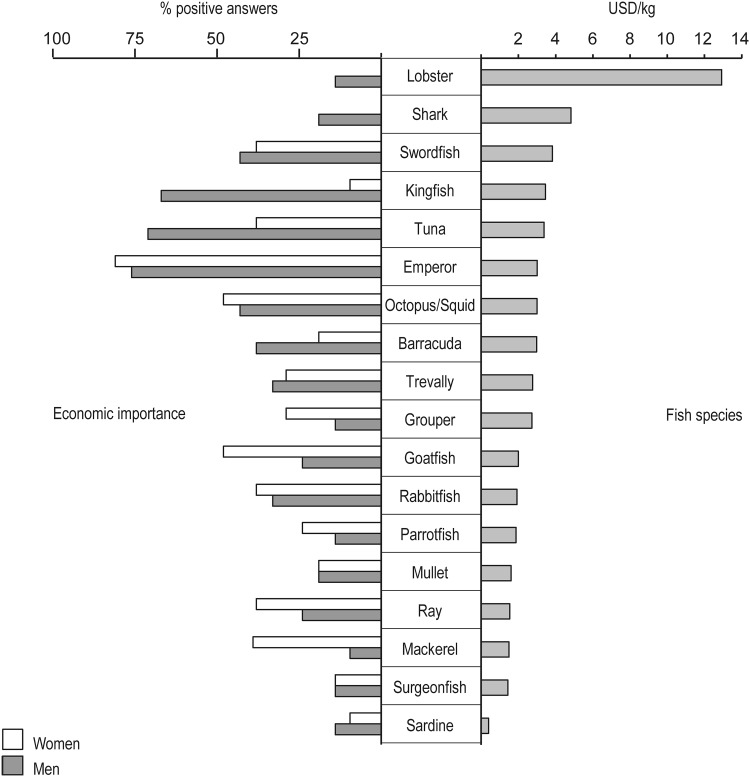
Average market prices for traded species (USD/kg) and economic importance for women (*n* = 21) and men (*n* = 21) traders, respectively (% positive answers)

All men interviewed sold fresh fish whereas many women sun-dried and/or smoked it before selling. The fish species reported to be of the highest economic importance for both men and women traders were Emperors (*Changu* in Swahili) (76 % of all men and 81 % of all women), which could be explained by a high demand but also by high natural fish abundances. Men tended to trade with a variety of medium- to high-value species such as tuna (71 % of all men), kingfish (67 %), octopus/squid (43 %), swordfish (43 %), barracuda (38 %), and trevally (33 %), while women’s income was mainly based on various medium- to low-value species such as octopus/squid (48 %), goatfish (48 %), mackerel (38 %), and rabbitfish (38 %). No women were involved in trade with shark and lobster, which are the two single most expensive species on the Zanzibar fish market, compared to 19 and 14 %, respectively, of the men traders (Fig. 4). All traded species with family names, English and Swahili are found in Electronic Supplementary Material, Table S3. The reasons given for choosing one species over another were linked to financial capital, availability, customers and access to cold storing facilities.

### Contacts and Organizations

#### Contacts

Both women and men expressed a wish to trade with hotels and restaurants but this requires contacts within the industry. Whereas 38 % of the men traders had contacts in hotels and/or restaurants, not a single woman did (Fig. 3c). This inequality was reported to be due to women’s lack of time to establish contacts, but also a result of the cultural setting and the common perception in Zanzibar that tourism-related work is unsuitable for women. However, apart from contacts within the tourism industry, about half of both the men and women traders had daily contact with other traders, fishers, or the auctioneer as a way to exchange information about, e.g., fish landings and demand. Further, all traders were asked about their relation to the former Department of Fisheries and Marine Resources, the institution responsible for fisheries management in Zanzibar. From the group, two women and eight men reported to have contact with the Department but most traders requested collaborations and information exchange.

#### Membership in Fish Trading Associations

Does an increasing number of women traders lead to a higher participation rate in formal organizations? This study found that there are no formal fish trading associations in Zanzibar. Instead, some men traders were members of the village’s fisheries committee, but since fishermen and traders have different interests (capture fisheries problems vs. market interests) this was reported to be unsatisfying. In one of the main markets in Zanzibar Town, 25 men had formed a group where they discussed fish trade-related issues, shared experiences and borrowed money from each other. This kind of self-organized group was present among men traders in other places around the island as well. No women were involved in the official fisheries committee neither in any kind of self-organized group. Most women respondents had no “free time” to spend on organizing and many of them stated a lack of arenas for discussion. However, there was an overall wish for organizations with focus on their needs and interests, but help from the Government was requested since “traders are just grassroot people and cannot organize without assistance” (Interview with woman trader in Mikunguni 2012). A minority of the traders were reluctant to organizations since “people are not trustworthy” (Interview with woman trader in Mwanakwerekwe 2012) and “everybody should run their own business” (Interview with woman trader in Malindi 2012).

### Perceptions and Experiences

#### Life Improvements and Equality

The majority of the respondents identified themselves as fish traders with high levels of job satisfaction (Fig. 3d). According to the respondents, society perceives fish trade as a high status activity; being a fish trader carries more prestige and greater economic status than being a fisher, seaweed farmer, or gleaner. The two main attributes for high job satisfaction were the sense of importance and recognition among people in their respective community. Another central factor was income. The majority of the interviewees said that fish trade has improved their living standards. The benefits included increased income and protein intake in the household, and many traders had been able to send their children to school, renovate houses and contribute to funerals, weddings, and loans. A majority of the traders also provided fish to people worse off than themselves, which increased their social status. In addition, most respondents reported gender equality at the auction sites. “What matters to succeed is a person’s capital and not whether he or she was born a man or a woman”. However, a large number of the men were worried about the increasing number of women traders and reported how women generally benefitted more from microcredit, which allowed them to buy higher quantities of fish. Women traders, on the other hand, found it difficult to compete with men on the auction site because men traders generally have higher capital, more experience and a broader contact net. From a vulnerability perspective, most traders reported not to have sufficient economic resources to cope with fluctuating markets (Fig. 3d). The women were mainly in need of credit and freezers to improve their business, withstand price hikes and cope with decreasing fish stocks. Similarly, most men requested credit but also a motorcycle to reduce time spent on local buses. Nevertheless, the overall attitude towards fish trade was very positive, especially among women who felt a sense of independence and more self-confident, due to an increased income and family well-being.

## Discussion

### Feminization of Fish Trade?

The first question posed was whether or not men still dominate this activity or if fish trade is slowly turning into a typical women profession, similar to what has been observed in other activities in Zanzibar such as seaweed farming. For example, men were initially engaged in seaweed farming but as income turned out to be very low in relation to workload, most men left the activity and returned to fishing; today 90 % of Zanzibar’s seaweed farmers are women (Fröcklin et al. 2012). Put in a fish trade context, decreasing stocks and smaller sizes of fish may open up space for women, whereas men would engage in other activities due to reduced profits. However, the results show that this does not seem to be the case in Zanzibar and the number of both men and women traders has increased, which is linked to the lack of livelihood options, but may also be a result of the financial crisis. However, the active involvement of women in an activity traditionally associated with men has the potential to challenge existing gender roles and empower women, and whether or not this is happening will be discussed below.

### To What Degree Has Fish Trade Led to Women Empowerment?

The results show that women traders had a higher educational level but, regardless of education, the women surveyed shared less access to profitable markets and mobility, financial capital, contacts and organizations, and high-value fish species. However, trading with less valuable fish seems to have some advantages; it requires less capital and has a ready market due to high local consumption. This could probably explain why most men traded with both commercially valuable species and species of lower value. By diversifying the customer base, traders may be less vulnerable to fluctuating prices and demand. However, most traders in this study had regular customers and bought fish according to the orders whereas other agreed upon, from day to day, what species to buy so as to minimize competition over customers. Men’s greater access to finance, profitable markets and customers, contacts, and commercially valuable fish resulted in income differentials. This shows that access to initial capital is a key factor for income enhancement, which in turn increases quality of life. However, since the women generally lacked own savings there are clear links to gender; women’s access to start-up capital is very much restricted by traditional gender roles in which women are associated with household duties and childcare. Reproductive work is unpaid work and thus makes it hard for women to accumulate start-up capital and gain experience. Also, lack of free time reduces the chances to network and establish contacts much needed to access more profitable markets. In addition, microcredit and loans do not seem to provide money enough to trade with high-value species or higher fish volumes required for a substantial income increase. These types of gender inequalities have been observed in other places as well (e.g., Medard et al. 2002; Tindall and Holvoet 2008) and not only reduce women’s access to fisheries resources, but also limit their bargaining power in case of price shocks or reduced fish landings. Nevertheless, due to the obviously commercial character of the activity, the average income for all fish traders irrespective of gender was quite high. A fish trader generally earns more than people involved in other coastal activities such as for example women invertebrate gleaners (0.5–0.6 USD per day) (Håkansson et al. 2012) and seaweed farmers (1 USD per day) (Fröcklin et al. 2012).

Despite of all problems and disadvantages illustrated above, women have still managed to enter a typical men-dominated arena. Hypothetically, a higher number of women could bring about change, not only by increasing the total household income and thus reducing poverty, but also women’s social and economic status. However, women’s greater involvement in productive work has not changed basic time-consuming activities associated to women’s reproductive role; for example, no women were involved in any type of fisheries organization, which was largely due to household responsibilities but also the lack of organizational skills. Unlike Zanzibar, there are places, such as for example Ghana, where women do belong to fish trader associations (Walker 2002), but membership in formal or informal fisheries associations and cooperatives is still more prevalent among men. Also, a higher number of women members may not per se increase women’s status unless the existing norms and structures change to accommodate women’s interests. Strong traditional norms and values in society may constrain women from actively participating in decision making. For example, in Mali many women traders are members of the traders’ association but they are disadvantaged by the social setting (Tindall and Holvoet 2008). This seems to be a general trend in many developing countries. Also, in many parts of Africa women have been involved in fishing through ownership, processing, and fish trade (Geheb et al. 2008; Béné et al. 2009), but most fishing communities are still dominated by men (Rangan and Gilmartin 2002). Thus, women’s efforts to improve their situation have placed them in men’s action arena, without necessarily challenging traditional gender roles. There are also examples of how women have addressed the poverty situation through agency and social entrepreneurship, and by doing so, also male subordination (Onyango and Jentoft 2011). Clearly, there are ways for women to break out of their traditional spatial boundaries and overcome the socially constructed roles ascribed to women and men, but in Zanzibar this process seems to lag behind or be slower in character. This demonstrates how well-rooted gender roles can be in the social and cultural setting of a specific location. Nevertheless, despite women’s limited power to economic and social resources, as well as decision making authority and organization, women traders reported high levels of job satisfaction, which is a common feature among fishermen (Pollnac et al. 2001; Allison and Horemans 2005), fisherwomen, fish traders, and processors (Appleton 2000). They further stated an increased level of self-esteem and awareness due to improved business skills. In conclusion, fish trade may have led to some level of empowerment but there is still a long way to go to diminish existing gender inequalities.

### Gender Sensitivity and Adaptive Management

Due to an increasing number of traders, and particularly women, policy and management must address the gender dimension to include the interests and needs as well as environmental impacts of both men *and* women. We therefore suggest an adaptive fisheries management approach. The first step in adaptive management processes is to identify problems and desired goals, and call for the development of suitable policy. Next is the implementation of policy and monitoring of results. According to Walters (1986) this process is iterative and offering not only the potential to engage different groups but also the opportunity for them to learn from each other. At present, Zanzibar’s fisheries management lack (i) research about the role of women in the fish value chain, (ii) gender-disaggregated data, and (iii) inclusion of these two in policy and management. The analysis of formal documents such as the Fisheries Act (Act 7 2010) (RGZ 2010) showed that the focal point of the Department of Fisheries Development (DFD), and its sub-sections such as deep-sea fishing and artisanal fisheries, is primarily on men-dominated capture fisheries and export whereas very little attention is drawn to local domestic fish trade. This clearly answers the question whether or not Zanzibar’s fisheries management and policy is gender sensitive. To overlook typical women activities like gleaning of invertebrates in the intertidal areas (Nordlund et al. 2010; Håkansson et al. 2012), or women traders’ activities, results in problem and goal identification since information is based on some sources only. Further, the lack of information available on fish traders reveal that they operate on an open informal market, without clear rules, control or taxation. This type of informal trade is rather common in developing countries throughout Africa but also in other places such as Mexico (Pedroza 2013). At present Zanzibar’s fisheries management seems not to be flexible and responsive enough to changing conditions, i.e., increase in traders and gender inequalities in the fish value chain, and there is no link between traders and formal institutions. Clearly, some of the most fundamental elements in adaptive management are missing.

### Implications for Policy Interventions

This study raises some important issues and should act as a stepping stone towards policy interventions. So, what is the way forward? First, one essential step is to collect statistics about the number of traders, sex, finance, traded species, and so on. Second, the establishment of fish trading organizations is essential to gather information about fish traders’ needs, problems, and interests. These types of organizations could be promoted by the Government and work as a platform for men and women to discuss common or different issues related to fish trade, and share knowledge and ideas while empowering women. Third, arenas that facilitate the formation of self-organized groups are needed and can act as a catalyst for better management and conservation. Arguably, a constant flow of fisheries resources should be in the traders’ interest. The creation of such meeting arenas could also lead to collaborations and exchange of ideas in the development of policies. Four, to facilitate the implementation of the recommendations above one could use existing structures and institutions. For example, statistics could be collected by increasing the number of monitoring agents (*Bwana Dikos*) already present at each market. In addition there are some experiences already gained in trying to organize seaweed farmers. Five, and most importantly, gender *must* be mainstreamed in all steps of fisheries management and policy making. We argue that Zanzibar’s fisheries management would benefit from an adaptive approach, which must be informed by extensive learning about the changing environment and people, both men and women, who depend upon it for daily survival, along with participation and an active dialogue throughout the process.

## Electronic supplementary material

Below is the link to the electronic supplementary material.

Supplementary material 1 (PDF 156 kb)
