# Supplementary material for: Fish Traders as Key Actors in Fisheries: Gender and Adaptive Management
Source: Ambio. 2013 Nov 9;42(8):951–62. doi: 10.1007/s13280-013-0451-1 (PMC3824874; doi:10.1007/s13280-013-0451-1)
Supplement: Supplementary file 1 — Supplementary material 1 (PDF 156 kb) [file 13280_2013_451_MOESM1_ESM.pdf]

AMBIO

Electronic Supplementary Material

Fish Traders as Key Actors in Fisheries: Gender and Adaptive Management

Sara Fröcklin, Maricela de la Torre-Castro, Lars Lindström, Narriman S Jiddawi

Appendix S1 Interview form

ID

Gender roles in fish trading, Zanzibar 2012

Supervisor.. Date and village....................................

Interpreter..

I. General information

- Respondent's name
- Age in years (or approximation)
- Marital status (married, single, divorced)
- Husband has other wives? (Yes or no and how many)
- How many children do you have? Boys/girls
- Education: □ no education □ Quran □ primary □ secondary □ other higher
- How many persons live permanently in this household?
- Who is the head of the household?
- Health status (good/medium/bad)

II. "A typical day as a fish trader"

Please tell me about a normal day as a fish monger, what do you do? What is the routine at home before going to the market?

- How do you get to the market?
- Is it always the same market?
- What kind of money do you use? Do you have a special pot of money just for fish trade? Do you get money from someone else? Explain.
- How do you decide where to go?

- How do you decide what fishes to buy?

III. Understanding the market: the broad picture

- How does this activity works in terms of money?
- Where do you get the money to buy fish?
- Do you have a specific pot of money used just to buy fish? From hotels? From microcredit or other?
- How do you handle the money you get from daily fish trade? For example, you get 30,000 TSH in your hand today, what do you do later? Do you put 30,000 TSH in the “pot” or you take 25,000 TSH in the pot and 5,000 TSH for family expenses? How do you do? Please explain!

IV. Equipment and materials (yes/no and 1. Not important 2. Medium 3. Very important)

**Equipment**	**Women yes/no**	**Importance 1-3**	**Men yes/no**	**Importance 1-3**
Cell phones				
Bicycle				
Motorcycle				
Freezer				
Fridge				
Baskets				
Knives				
Table				
Calculator				
Ice				
Scissors				
Bags				

V. Economic resources

- How much money you buy fish for per day?
- Is it always the same amount or is there variation?
- And why?
- How much money per day approx. do you get from fish trade?
- How much of that money you take as income?
- How much of that money you reinvest in the pot? (link to part III understanding the market)
- Besides buying fish, what other expenses associated with this activity do you have?
- Do you have any microcredit or other forms of support, loans?

VI. Knowledge

- How do you decide what fish to buy?
- How do you know in which market to buy?
- How do you know to whom to sale fish?
- Who are your best customers?
- Would you like to have other customers?
- Do you know how other traders work?
- Alone or with assistance from friend, relative, family or other?
- Do you gain “tips” and/ or experience from other traders/friends/family?
- Do you know what species are best to get good money?
- If so, what species?
- Do you know what species are best to get good food?
- If so, what species and why?

VII. Contacts

- Do you have any contacts in hotels, restaurants?
- Other contacts (for example friends, family) that is important to you or help you with this activity?

- Do you get assistance during the auction?
- From whom?
- Why do you need assistance?
- Do you have any contact with the Department of fisheries and marine resources?
- If no, have you heard of it or other fish traders that are in contact with this department?

VIII. Organization

- Do you belong to any organization?
- Cooperative?
- Fish trading association?
- NGO?
- If yes, what do you gain from the membership?
- If no, have you heard of any of these organizations? Or other?
- Do you know if other fish mongers are involved?
- Why are you not involved in any organization, cooperative or other?
- What would you need to get involved? Meeting places? Time?
- How would men react of a cooperative that increased women's incomes?
- If they would not be happy, how would you go about?

IX. Household duties and decision making

- What are your main responsibilities in the household?
- How much time to you spend on different household chores? In relation to other family members?
- How much free time do you have? Compared to your husband/wife?
- How much time do you spend on fish trade?
- Would you spend more time on fish trade if you had less household work?
- What issues and resources in the household do you decide over? What else would you like to decide?

X. Markets and Mobility

- Are you happy with your market and with your buyers?
- Or would you like to buy and sell fish somewhere else?
- Or would you like to have other buyers?
- If yes, what kind of buyers would you like?
- How do you cope with a bad market (fewer buyers, decreasing fish, and high price)?
- Do you have the possibility to go to another market if this one is bad?
- If so, what kind of transport do you use and how often do you go?
- How accessible are the roads, good/bad?

XI. Perceptions and experiences

- Would you identify yourself as a fish trader?
- Or something else?
- Are you satisfied/proud of being a fish trader?
- Why/why not?
- Is fish trading high status in the society?
- What effects has fish trade had on your life (e.g. income, increased decision making power in the household)?
- On the community (increased decision making power, poverty alleviation, and development)?
- Do you feel like you have the resources to cope with changes in the fishery and/or the market (decreasing fish stocks, increasing prices in fish, fuel wood, oil etc.?)
- What are your strategies to handle those?
- What are your dreams and hopes for the future?

Species/local	Buy yes/no	Economic	Food
Tasi/Rabbitfish			
Pono/Parrotfish			
Changu/Emperor			
Chewa/Grouper			
Mkundaji/Goatfish			
Puju/Surgeon			
Mkizi/Mullet			
Dagaa/Sardine			
Vibua/Mackerel			
Kole kole/Trevally			
Jodari/Tuna			
Nduaro/Swordfish			
Nguru/Kingfish			
Mzia/Barracuda			
Papa/Taa/Shark/Ray			
Pweza/Ngisi/Octopus/Squid			
Kamba/Lobster			
Wengineo/Others			

Table S2 Market, transport means and average daily distance

Gender	*Auction*	*End market*	*Transport*	*Distance (km)*
W	Chwaka, Uroa	Town, food kiosks*	Dala dala	60
W	Darajani	Town*	Dala dala	5
W	Malindi	Mwanakwerekwe**	Dala dala	10
W	Malindi	Mwanakwerekwe **	Dala dala	10
W	Malindi,	Mwanakwerekwe **	Dala dala	10
W	Darajani	Mwanakwerekwe **	Dala dala	10
W	Malindi	Town*	Dala dala	5
W	Malindi	Town*	Walk	3
W	Marumbi	Town*	Dala dala	60
W	Marumbi	Bambi*	Dala dala	30
W	Mkokotoni	Amani*	Dala dala	70
W	Mkokotoni	Mahonda*	Dala dala	30
W	Malindi, Darajani	Mikunguni*	Dala dala	20
W	Malindi	Mikunguni*	Dala dala	20
W	Malindi	Mikunguni*	Dala dala	10
W	Malindi	Mikunguni*	Dala dala	10
W	Nungwi	Amani*	Dala dala	100
W	Nungwi	Mikunguni*	Dala dala	100
W	Uroa	Jendele*	Dala dala	30
W	Uroa	Amani *	Dala dala	60
W	Uroa, Chwaka	Town*	Dala dala	60
M	Chwaka	Koani*	Bicycle	30
M	Chwaka	Fuoni**	Bicycle	40
M	Darajani	Darajani ***	Dala dala	6

M	Darajani	Darajani***	Dala dala	6
M	Malindi	Mwanakwerekwe **	Dala dala	10
M	Malindi	Malindi***	Dala dala	6
M	Malindi	Malindi***	Walk	6
M	Marumbi	Town *	Bicycle/dala dala	60
M	Matemwe	Matemwe*, Town ****	Walk/dala dala	0-80
M	Matemwe	Malindi****	Dala dala	80
M	Matemwe	Matemwe ***	Walk	0
M	Mkokotoni	Town**	Dala dala	5
M	Mkokotoni	Mkokotoni*	Dala dala	5
M	Nungwi	Nungwi*****	Walk	0
M	Nungwi	Darajani ***	Bicycle	100
M	Nungwi	Darajani *****	Walk/dala dala	100
M	Nungwi	Malindi/ Darajani ****	Walk/dala dala	100
M	Nungwi	Malindi/ Darajani****	Walk/dala dala	100
M	Nungwi	Malindi/Darajani****, Nungwi*****	Walk/dala dala	100
M	Uroa	Uroa*****	Walk	0
M	Uroa	Uroa*	Walk	20

* street vendor ** small market vendor *** Town market vendor ****auction ***** hotel

Table S3 Traded species (English and Swahili) from high to low value

Family	*English name*	*Local name (Swahili)*
Palinuridae	Lobster	Kamba
Carcharhinidae	Shark	Papa
Xiphiidae	Swordfish	Nduaro
Scombridae	Kingfish	Nguru
Scombridae	Tuna	Jodari
Lethrinidae	Emperor	Changu
Cephalopoda spp.	Octopus	Pweza
Cephalopoda spp.	Squid	Ngisi
Sphyraenidae	Barracuda	Mzia
Carangidae	Trevally	Kole kole
Serranidae	Grouper	Chewa
Mullidae	Goatfish	Mkundaji
Siganidae	Rabbitfish	Tasi
Scaridae	Parrotfish	Pono
Mugilidae	Mullet	Mkizi
Dasyatidae	Ray	Taa
Scombridae	Mackerel	Vibua
Acanthuridae	Surgeonfish	Puju
Clupeidae	Sardine	Dagaa
